# Supplementary material for: Controlled Release of Bone Morphogenetic Protein-2 Augments the Coupling of Angiogenesis and Osteogenesis for Accelerating Mandibular Defect Repair
Source: Pharmaceutics. 2022 Nov 7;14(11):2397. doi: 10.3390/pharmaceutics14112397 (PMC9699026; doi:10.3390/pharmaceutics14112397)
Supplement: Supplementary file 1 [file pharmaceutics-14-02397-s001.zip › pharmaceutics-1958900-supplementary.pdf]

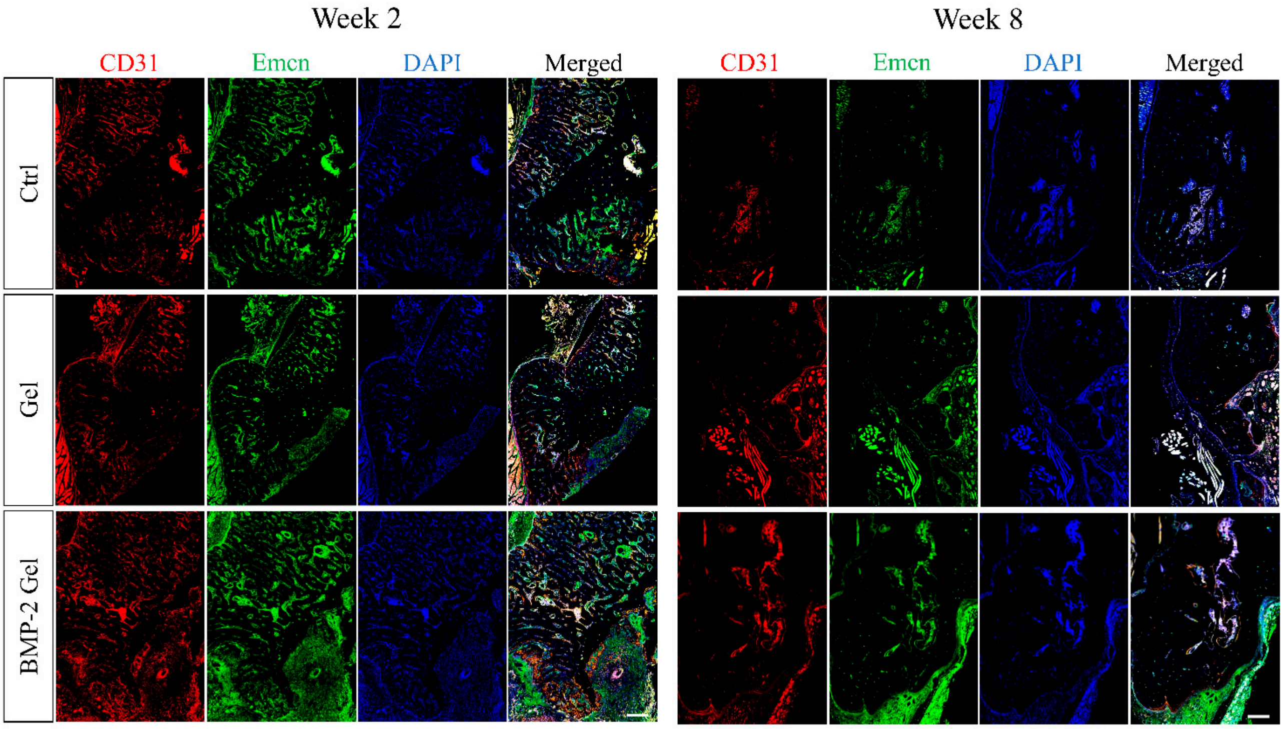

Supplementary Figure S1 Immunofluorescent staining of type H vessels (CD31+ Emcn+) in the mandibular bone. Scale bar, 250 μm.

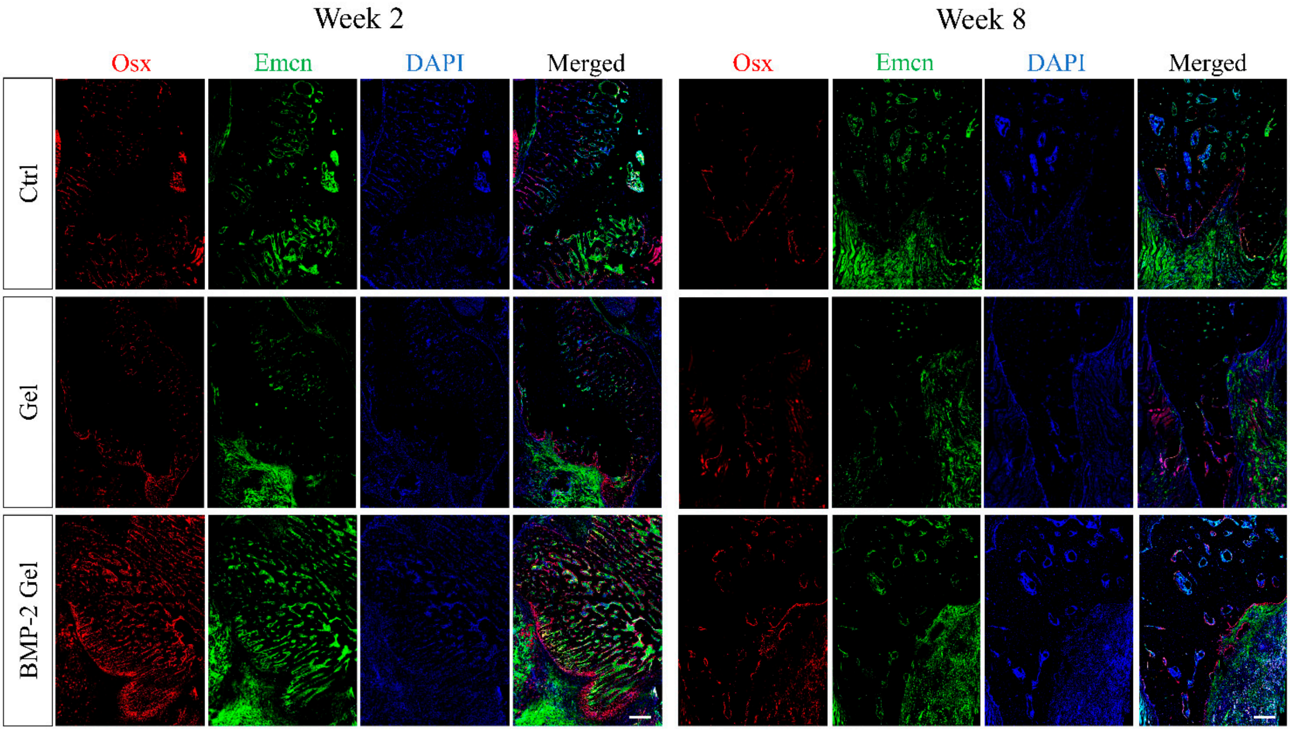

Supplementary Figure S2 Immunofluorescent staining of Osx+ osteoprogenitor cells and Emcn+ vessels in the mandibular bone. Scale bar, 250 μm.

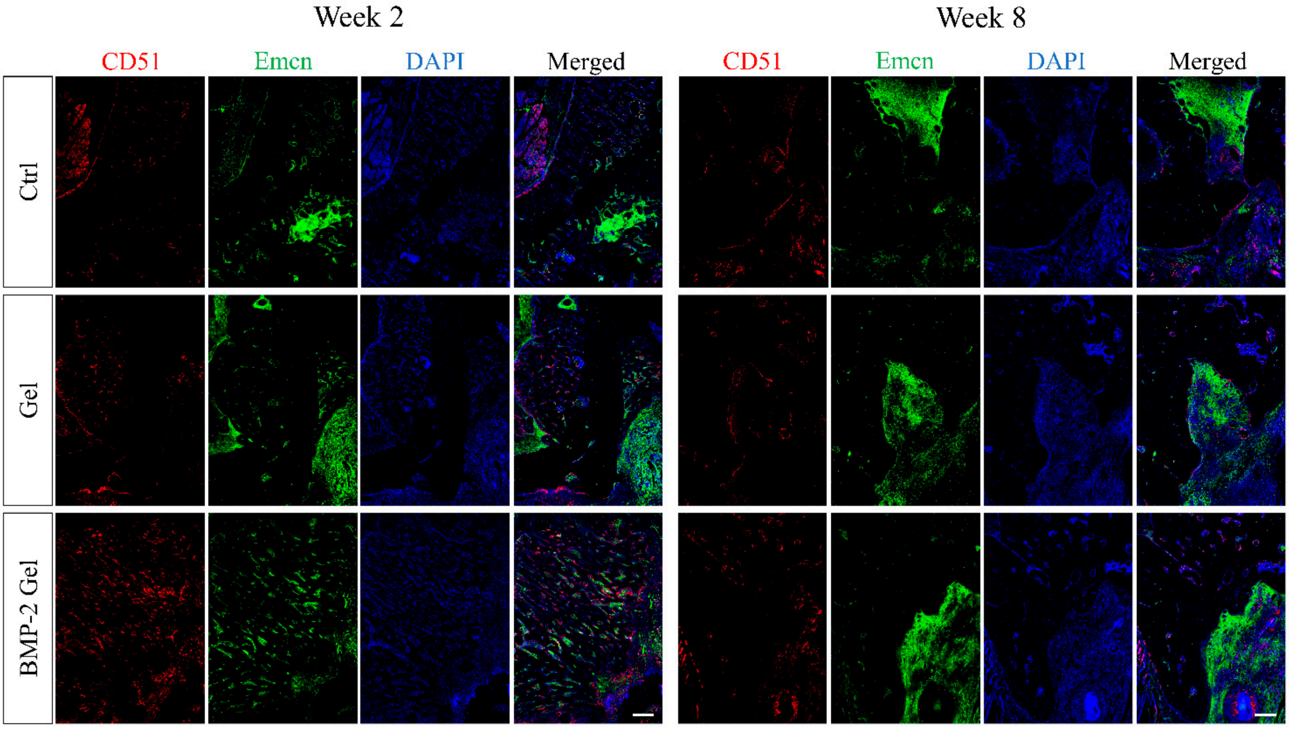

Supplementary Figure S3 Immunofluorescent staining of CD51+ cells and Emcn+ vessels in the mandibular bone. Scale bar, 250 μm.

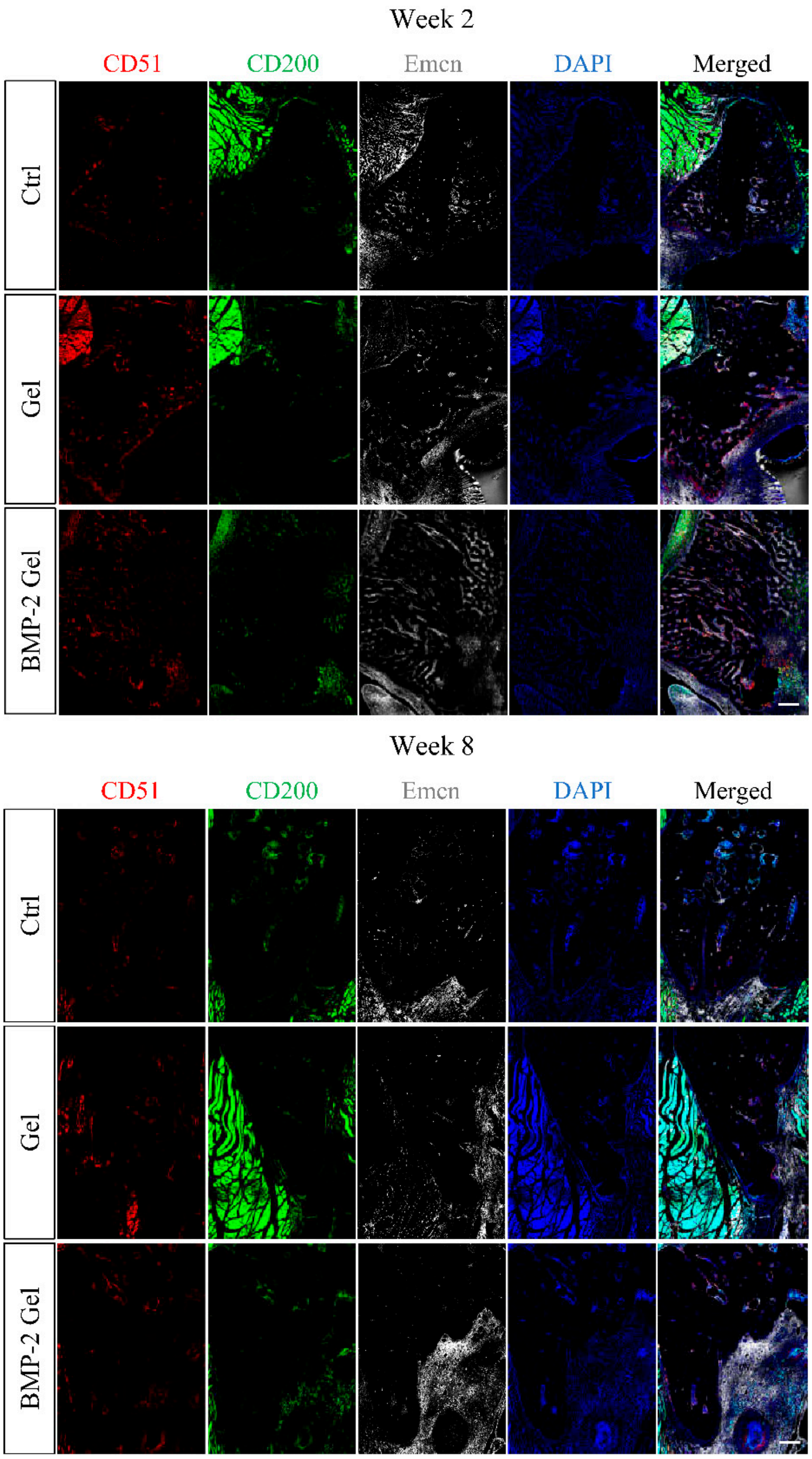

Supplementary Figure S4 Immunofluorescent staining of CD51+ CD200+ SSCs and Emcn+ vessels in the new bone. Scale bar, 250 μm.

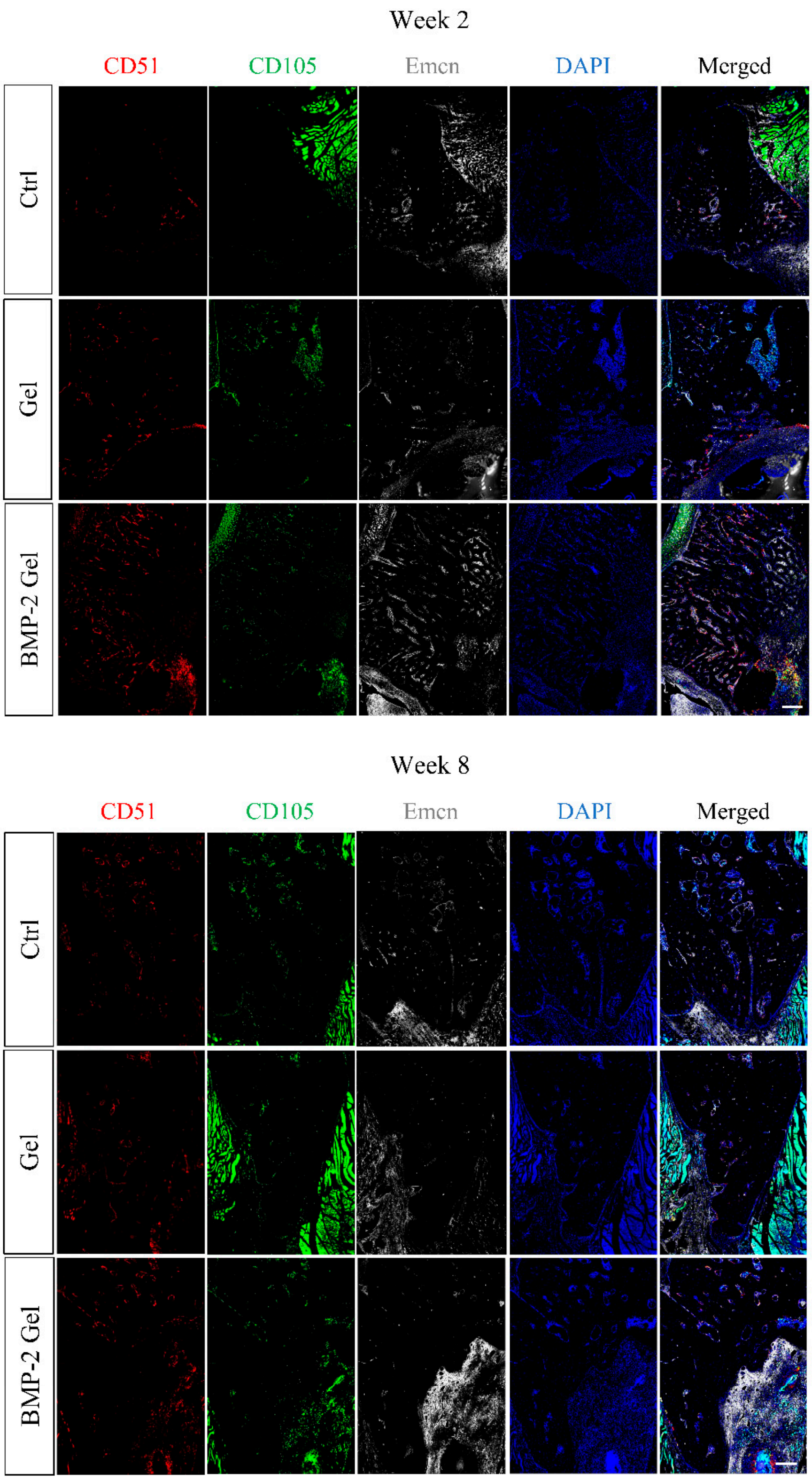

Supplementary Figure S5 Immunofluorescent staining of CD51+ CD105+ SSCs and Emcn+ vessels in the new bone. Scale bar, 250 μm.

Supplementary Table S1 Information of antibodies used in this study

**Antibodies**		
CD31	R&D system, af3628	1:150
Endomucin (Emcn)	Santa Cruz, sc65495	1:150
CD51	ThermoFisher, MA5-32195	1:200
CD200	Abcam, ab34087	1:100
CD105	Abcam, ab252345	1:200
Osterix (Osx)	Abcam, ab22552	1:200
Anti-goat Alexa Fluor 546	ThermoFisher, A11056	1:400
Anti-rat Alexa Fluor 488	ThermoFisher, A21208	1:400
Anti-rabbit Alexa Fluor 647	Abcam, ab150155	1:400
